# Supplementary material for: Enhanced critical-current in P-doped BaFe2As2 thin films on metal substrates arising from poorly aligned grain boundaries
Source: Sci Rep. 2016 Nov 11;6:36828. doi: 10.1038/srep36828 (PMC5104974; doi:10.1038/srep36828)
Supplement: Supplementary Information [file srep36828-s1.pdf]

Supplementary information for "Enhanced critical-current in P-doped $BaFe_2As_2$ thin films on metal substrates arising from poorly aligned grain boundaries"

Hikaru Sato[1], Hidenori Hiramatsu[1,2], Toshio Kamiya[1,2], and Hideo Hosono[1,2]

[1]*Laboratory for Materials and Structures, Institute of Innovative Research, Tokyo Institute of Technology, Japan*

[2]*Materials Research Center for Element Strategy, Tokyo Institute of Technology, Japan*

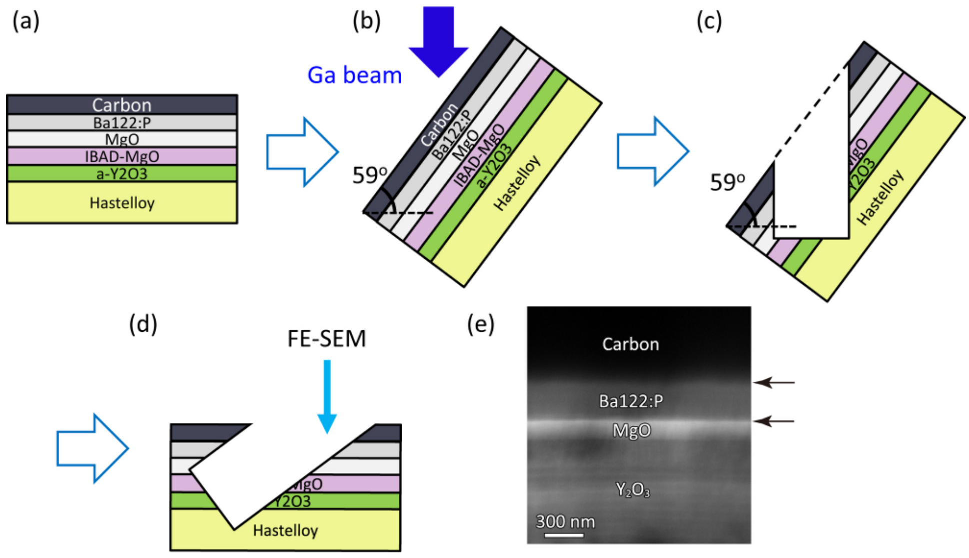

Figure S1. Determination procedure of the thickness of the Ba122:P film on the IBAD-MgO substrate. (**a**) Schematic cross-sectional illustration of stacking structure before FIB treatment. (**b**) Configuration of the sample and a gallium-ion beam in FIB system. (**c**) Schematic cross-sectional image after FIB etching processing. (**d**) The sample was transferred to the FE-SEM system to observe the cross section of the FIB-processed position. (**e**) An example of the cross-sectional FE-SEM image of a sample. The horizontal arrows indicate the upper and the bottom interfaces of the Ba122:P film. From this image the thickness is determined to be 185 nm using the observed film layer thickness of 308 nm and the observation angle of 31° (185 nm = 308 nm × tan 31°). This procedure was applied to all the samples that we measured transport properties.

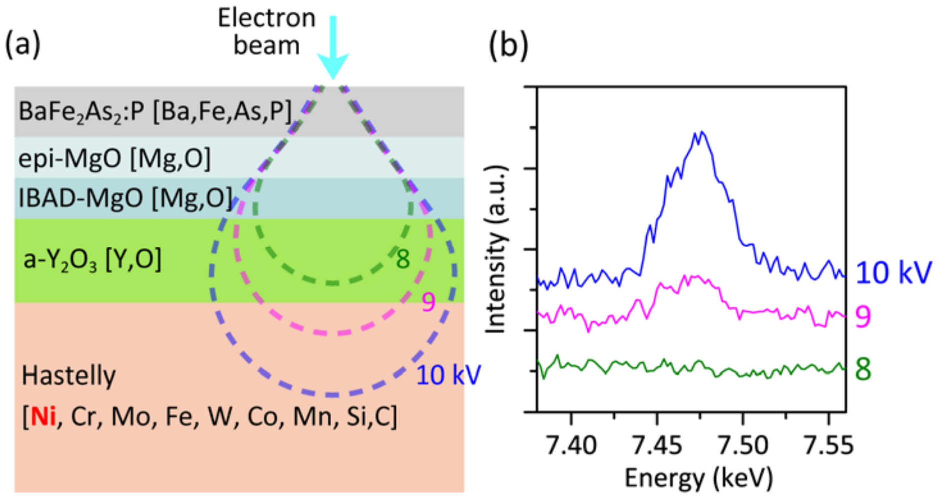

Figure S2. Determination procedure of the acceleration voltage of the electron beam in the electron-probe microanalyzer. (**a**) Schematic cross-sectional illustration of the sample and the penetration of electron at 8 – 10 kV. (**b**) The Ni Kα spectra at 8 – 10 kV. Based on these spectra, we performed the chemical composition analysis at 8 kV.
